# Berberine-Functionalized Graphene Oxide Nanocomposite for Enhanced Corrosion Protection of Epoxy-Coated Copper in Marine Environments

**DOI:** 10.3390/ma19061080

**Published:** 2026-03-11

**Authors:** Hassane Lgaz

**Affiliations:** Innovative Durable Building and Infrastructure Research Center, Center for Creative Convergence Education, Hanyang University ERICA, 55 Hanyangdaehak-ro, Sangrok-gu, Ansan-si 15588, Gyeonggi-do, Republic of Korea; hlgaz@hanyang.ac.kr

**Keywords:** corrosion protection, graphene oxide, epoxy coating, copper, berberine, nanocomposite coating

## Abstract

This study introduces a novel anticorrosion coating for copper based on an epoxy matrix reinforced with a berberine-loaded graphene oxide (BBR@GO) nanocomposite. The BBR@GO was synthesized via a simple, non-covalent functionalization method, leveraging π-π stacking interactions between the planar berberine molecule and the graphene oxide surface. The successful loading of berberine was confirmed by Fourier-transform infrared (FTIR) spectroscopy, thermogravimetric analysis (TGA), and energy-dispersive X-ray spectroscopy (EDS). The BBR@GO nanocomposite was incorporated into an epoxy resin at 0.1 wt.% loading and applied to a copper substrate. The corrosion protection performance of the BBR@GO/EP coating was systematically evaluated in 3.5 wt.% NaCl solution for 27 days using electrochemical impedance spectroscopy (EIS) and potentiodynamic polarization (PDP). The BBR@GO/EP coating exhibited a total impedance of 5.31 × 10^8^ Ω·cm^2^ after 27 days, which was 17 times higher than the pure epoxy (EP) coating. The corrosion current density (i_corr_) was reduced to 2.59 × 10^−8^ A·cm^−2^, a four-fold decrease compared to the EP coating. Post-immersion analysis confirmed the excellent durability of the BBR@GO/EP coating and the retention of berberine within the matrix. The enhanced performance is attributed to the synergistic effect of the physical barrier provided by the well-dispersed GO nanosheets and the inhibitive action of the retained berberine molecules at the coating–metal interface.

## 1. Introduction

Copper and its alloys are essential materials in marine and industrial applications [1,2]. Their use is widespread due to excellent thermal and electrical conductivity. However, copper is susceptible to severe corrosion in aggressive environments like seawater [3,4]. Chloride ions in marine environments accelerate the degradation process significantly. This corrosion compromises the structural integrity and performance of copper components. Therefore, developing effective corrosion protection strategies is of paramount importance [5,6].

Organic coatings are a primary method for protecting metallic substrates from corrosion [7,8]. Epoxy resins are widely used as protective coatings. They offer strong adhesion, good chemical resistance, and effective barrier properties. However, epoxy coatings are permeable to corrosive species like water, oxygen, and chloride ions over long-term exposure [9,10]. Micropores and defects in the coating can also provide direct pathways for corrosive agents to reach the metal surface. This leads to localized corrosion and coating delamination [11,12].

Incorporating nanofillers into the epoxy matrix can significantly enhance its protective performance. Graphene oxide (GO) has emerged as a promising nanofiller for anticorrosion coatings [13]. Its two-dimensional lamellar structure creates a highly tortuous path for diffusing corrosive species. This “maze effect” significantly improves the barrier properties of the epoxy coating [14,15]. Several studies have demonstrated the effectiveness of GO in enhancing the corrosion resistance of epoxy coatings. For instance, Pourhashem et al. investigated the effect of GO loading on the corrosion protection of epoxy coatings on mild steel [16]. They found that a low loading of 0.1 wt.% GO provided the most significant improvement in barrier properties. Ramezanzadeh et al. further advanced this by using amino-functionalized GO (FGO) in an epoxy matrix [17]. Their work showed that covalent functionalization improved the dispersion of GO and enhanced the cross-linking density of the epoxy, leading to superior corrosion resistance. Recently, researchers have explored coating systems where GO functionalized by corrosion inhibitors. Yan et al. developed a GO-based nanomaterial loaded with 2-mercaptobenzothiazole (MBT) that provided both passive barrier protection and active, pH-responsive inhibitor release [18]. Similarly, Cui et al. used functionalized GO to carry 1-hydroxyethylidene-1,1-diphosphonic acid (HEDP), demonstrating a synergistic chemical inhibition and physical barrier effect in waterborne epoxy coatings [19].

While these studies show great promise, challenges remain. Covalent functionalization of GO often involves complex, multi-step synthesis procedures. Furthermore, many synthetic corrosion inhibitors loaded onto GO, such as benzotriazole or MBT, raise environmental concerns due to their toxicity. This has motivated a search for green, sustainable, and effective additives for GO-based coatings. Non-covalent functionalization of GO using π-π stacking interactions presents a simpler and more scalable alternative to covalent methods [20,21]. It preserves the intrinsic electronic structure of the GO lattice while allowing for the loading of functional molecules. Natural products with planar aromatic structures are ideal candidates for this approach.

Berberine (BBR) is a natural isoquinoline alkaloid extracted from plants of the Berberis species [22,23]. Its planar molecular structure makes it suitable for non-covalent attachment to the GO surface via π-π stacking. Berberine is also known to be an effective corrosion inhibitor for various metals, including copper, in acidic and neutral solutions [24,25,26]. Loading berberine onto GO nanosheets could create a simpler dual-functional nanocomposite. This composite would combine the excellent physical barrier properties of GO with the active inhibitive action of a green, naturally derived molecule.

This work introduces a novel anticorrosion system based on a berberine-loaded graphene oxide (BBR@GO) nanocomposite. The BBR@GO was prepared via a simple, non-covalent functionalization method. The nanocomposite was then incorporated into an epoxy matrix to create a protective coating for copper substrates. The objective of this study is to investigate the corrosion protection effect of the BBR@GO nanocomposite in a simulated marine environment (3.5 wt.% NaCl). The study employs electrochemical and surface characterization techniques to elucidate the protection mechanism. The novelty of this work lies in the use of a natural, green additive (berberine) to create a non-covalently functionalized GO-based nanocomposite for the enhanced corrosion protection of copper.

## 2. Materials and Methods

### 2.1. Chemicals

Graphene oxide powder (single layer, 99.3% purity) was purchased from US Research Nanomaterials Inc (Houston, TX 77084, USA). Berberine chloride was obtained from Sigma-Aldrich (St. Louis, MO, USA). A DGEBA-based epoxy resin, and its polyamide hardener were procured from Samhwa Paint Industrial Co., Ltd., Ansan-si, South Korea. Copper sheets (99.9% purity) were obtained from a local supplier. All other reagents and solvents were obtained from Duksan Pure Chemicals, Ansan-si, South Korea. All chemicals were of analytical grade and used as received without further purification. Deionized (DI) water was used throughout all experiments.

### 2.2. Preparation of Berberine-Loaded Graphene Oxide (BBR@GO)

The BBR@GO nanocomposite was prepared via a simple, room-temperature physisorption method driven by π-π stacking interactions. A GO dispersion was prepared by dispersing 200 mg of GO in a mixed solvent system of DMSO and deionized water (2:8, *v*/*v*), followed by dilution to a final volume of 100 mL. Separately, berberine was dissolved in DMSO and diluted with deionized water to obtain a 100 mL homogeneous solution. The berberine solution was then added to the GO dispersion, and the resulting mixture was sonicated for 30 min and subsequently magnetically stirred at room temperature for 24 h to promote effective functionalization. The pH was adjusted to 7.4. The BBR@GO nanocomposite was collected by centrifugation and washed repeatedly with deionized water through centrifugation–redispersion cycles. Finally, the purified nanocomposite was dried in a vacuum oven at 50 °C for 24 h to obtain a dry powder.

### 2.3. Preparation of Coatings

**Figure 1:**
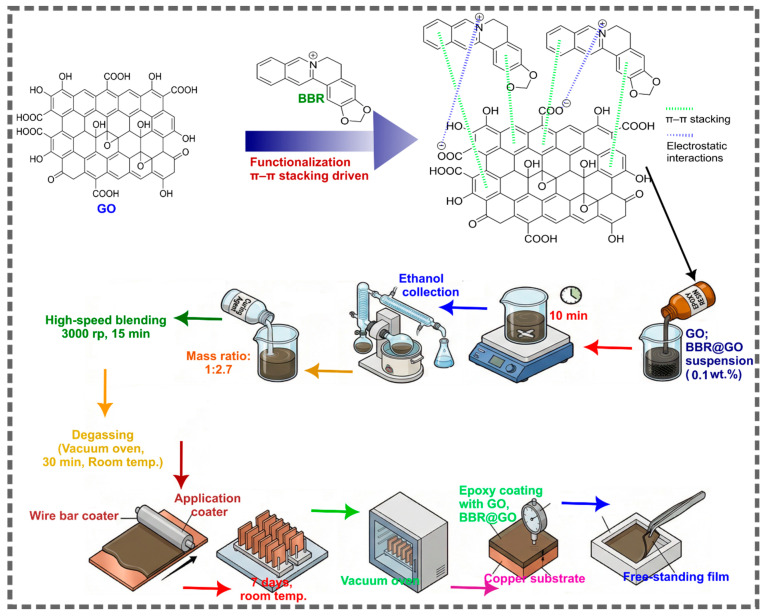
A schematic representation of the functionalization and coating preparation process.

Copper sheets (99.9% purity, 1 mm thickness) were cut into 2 cm × 2 cm coupons and sequentially abraded with 400, 800, 1200 and 2000 grit SiC paper. The abraded coupons were degreased with acetone. They were then rinsed with DI water and dried in a stream of hot air. GO or BBR@GO (0.010 g; 0.1 wt.% of the final formulation) was first dispersed in ethanol, after which 9.990 g of epoxy resin was added under stirring. After being carefully stirred for 10 min, ethanol was removed by rotary evaporation. The curing agent (epoxy/curing agent mass ratio = 2.7:1) was then added, and the mixture was thoroughly mixed using a high-speed blender at 3000 rpm for 15 min. Subsequently, the mixture was degassed in a vacuum oven at room temperature for 30 min to remove trapped air bubbles and was ready for the preparation of different coating samples. The reinforced epoxy coatings were applied onto copper substrates using a wire bar coater. The coated samples were air-dried at room temperature for 7 days and subsequently post-cured in a vacuum oven at 90 °C for 1 h. Two types of coating samples were prepared for different measurements. (1) Coated copper samples for electrochemical measurements. The coating thickness was measured using a film thickness gauge and from cross-sectional images obtained by SEM. (2) Free-standing films were obtained by casting the epoxy mixtures into PTFE molds followed by curing and were used for coating characterization. Pure epoxy, graphene oxide reinforced epoxy, and epoxy reinforced by BBR-functionalized GO were noted EP, GO/EP, and BBR@GO/EP, respectively. A schematic representation of the functionalization and coating preparation process is shown in Figure 1.

### 2.4. Adhesion Test

Adhesion of the coatings to the copper substrate was evaluated using the cross-cut tape test according to ASTM D3359, Method B [27,28]. A lattice pattern consisting of six cuts in each direction was scribed into the coating using a multi-blade cutter with 1 mm spacing. Pressure-sensitive tape was firmly applied over the lattice area. The tape was then removed by pulling it off at an angle of approximately 180° in a single, smooth motion. The adhesion was rated on a scale from 5B (no peeling or removal) to 0B (greater than 65% removal of the coating). Three replicate tests were performed for each coating type.

### 2.5. Material and Surface Analysis

Fourier-transform infrared (FTIR) spectroscopy was performed on a Perkin Elmer UATR Two (Waltham, MA, USA) equipped with an attenuated total reflectance (ATR) accessory. Spectra were recorded in the range of 400–4000 cm^−1^ with a resolution of 4 cm^−1^. Each spectrum was averaged over 32 scans. FTIR was used to characterize the functional groups of the prepared powders (GO, BBR, BBR@GO). It was also used to analyze the coating surfaces after immersion testing. Thermogravimetric analysis (TGA) was conducted using a simultaneous thermal analyzer (STA 600, TA instruments, New Castle, DE USA). Approximately 10 mg of each sample was heated from 30 to 800 °C at a constant heating rate of 10 °C/min. All measurements were performed under a nitrogen atmosphere with a flow rate of 20 mL/min.

The morphology and elemental composition of the nanocomposite powders and the coated surfaces were examined using a Hitachi S-4800 field emission scanning electron microscope (FESEM) (Hitachi, Ltd., Chiyoda City, Tokyo, Japan), after platinum (Pt) coating. The microscope was equipped with an energy-dispersive X-ray spectroscopy (EDS) detector. The accelerating voltage was set to 10 kV for imaging and 15 kV for EDS analysis. Powder samples were dispersed on carbon tape prior to analysis.

### 2.6. Electrochemical Testing

All electrochemical measurements were performed using a potentiostat/galvanostat autolab PGSTAT302N connected to a computer with NOVA 1.10 software. A conventional three-electrode cell configuration was employed. The coated copper coupon served as the working electrode. Carbon wire was used as the counter electrode. A saturated calomel electrode (SCE) served as the reference electrode. The electrolyte was a 3.5 wt.% NaCl aqueous solution prepared with analytical-grade reagents and DI water. Prior to each measurement, the open circuit potential (OCP) was monitored for 30 min to ensure a stable state.

Electrochemical Impedance Spectroscopy (EIS) measurements were conducted after 3, 9, 18, and 27 days of immersion. The maximum exposure time of 27 days was selected to provide a month-scale immersion assessment that is sufficient to capture the key evolution of barrier coatings in 3.5 wt.% NaCl, from the initial uptake stage to progressive aging, while maintaining good experimental reproducibility for EIS measurements and fitting. The chosen intervals (3, 9, 18, and 27 days) were designed to track early, intermediate, and late changes and to enable a clear performance comparison among EP, GO/EP, and BBR@GO/EP. Immersion tests were performed by continuously exposing the coated coupons to 3.5 wt.% NaCl in sealed containers at room temperature. At each time point, a coupon was briefly removed, mounted in the electrochemical cell containing fresh electrolyte, and EIS was recorded at steady open-circuit potential. The coupon was then returned to the immersion container to continue aging. The EIS spectra were acquired at the OCP with a sinusoidal perturbation amplitude of 10 mV. The frequency range was swept from 100 kHz to 10 mHz. The impedance data were fitted to appropriate equivalent electrical circuits using EC-Lab software V11.27. Potentiodynamic polarization (PDP) curves were recorded after 27 days of immersion. The potential was scanned from −250 mV to +250 mV relative to the OCP at a scan rate of 1 mV/s. The corrosion potential (E_corr_) and corrosion current density (i_corr_) were determined by Tafel extrapolation of the linear portions of the anodic and cathodic branches. The EIS data shown in the manuscript correspond to representative curves, and all electrochemical tests were performed on two independent specimens per condition.

## 3. Results and Discussion

### 3.1. Characterization of BBR@GO Nanocomposite

#### 3.1.1. FTIR Spectroscopy

**Figure 2:**
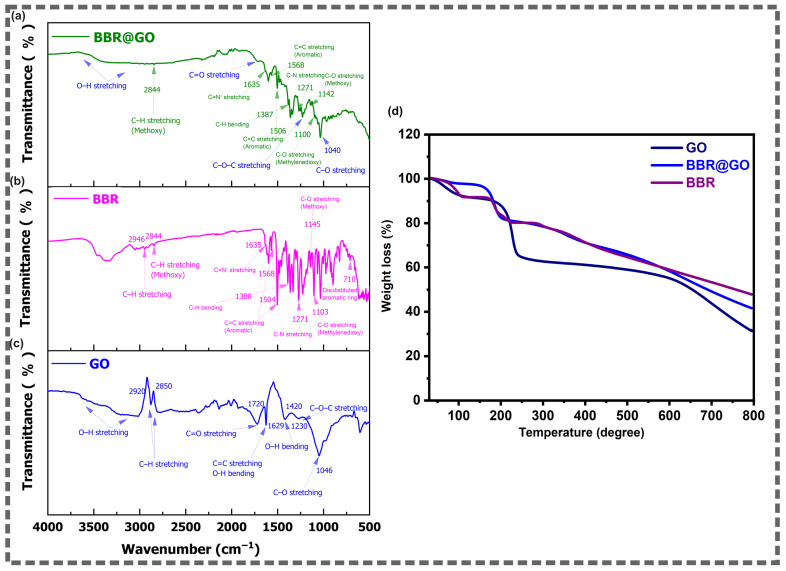
FTIR analysis of (**a**) BBR@GO, (**b**) BBR, and (**c**) GO, and (**d**) represents the TGA curves of GO and BBR@GO.

The FTIR spectra of pristine GO, pure berberine, and the BBR@GO nanocomposite are presented in Figure 2a–c. The spectrum of pristine GO (Figure 2c) exhibits the characteristic absorption bands of chemically oxidized graphene. A broad absorption band in the 3000–3700 cm^−1^ region is attributed to O–H stretching vibrations of hydroxyl groups and adsorbed water molecules [29]. Two bands at 2920 and 2850 cm^−1^ correspond to asymmetric and symmetric C–H stretching vibrations of residual aliphatic carbon, respectively [30]. A strong absorption at 1720 cm^−1^ is assigned to C=O stretching of carboxyl groups located at the edges of the GO sheets [29,31]. The band at 1629 cm^−1^ is attributed to the overlapping contributions of C=C skeletal stretching of the sp^2^ carbon network and O–H bending of adsorbed water [32]. The peak at 1420 cm^−1^ corresponds to O–H bending vibrations. The absorption at 1230 cm^−1^ is assigned to C–O–C stretching of epoxide groups on the basal plane [33]. The band at 1046 cm^−1^ is attributed to C–O stretching of alkoxy groups [33].

The FTIR spectrum of pure berberine (Figure 2b) displays a rich fingerprint region characteristic of its isoquinoline alkaloid structure. The bands at 2946 and 2844 cm^−1^ are assigned to aliphatic C–H stretching and methoxy C–H stretching vibrations, respectively [34]. The absorption at 1635 cm^−1^ corresponds to the C=N^+^ stretching vibration of the iminium group [34,35]. Two bands at 1568 and 1504 cm^−1^ are attributed to aromatic C=C stretching vibrations of the isoquinoline ring system [34]. The peak at 1388 cm^−1^ is assigned to C–H bending deformation [36]. The band at 1271 cm^−1^ corresponds to C–N stretching of the tertiary amine linkage [37]. The absorptions at 1145 and 1103 cm^−1^ are attributed to C–O stretching of the methoxy groups and the methylenedioxy bridge (–OCH_2_O–), respectively [38]. The band at 710 cm^−1^ can be assigned to the out-of-plane C–H bending of the disubstituted aromatic ring.

The FTIR spectrum of the BBR@GO nanocomposite (Figure 2a) displays a clear superposition of the characteristic peaks of both GO and berberine. The broad O–H stretching band in the 3000–3700 cm^−1^ region remains present. The methoxy C–H stretching band of berberine appears at 2844 cm^−1^. The C=O stretching band of GO is observed as a shoulder near 1720 cm^−1^. The diagnostic iminium C=N^+^ stretching band of berberine is resolved at 1635 cm^−1^. The aromatic C=C stretching bands of berberine are observed at 1568 and 1506 cm^−1^. The C–H bending band appears at 1387 cm^−1^. The C–N stretching band of berberine is detected at 1271 cm^−1^. The methoxy C–O stretching and methylenedioxy C–O stretching bands are observed at 1142 and 1100 cm^−1^, respectively. The GO alkoxy C–O stretching band appears at 1040 cm^−1^.

The simultaneous presence of the berberine iminium C=N^+^ peak at 1635 cm^−1^ and the GO carboxyl C=O peak at ~1720 cm^−1^ confirms the coexistence of both components in the nanocomposite. The slight shifts observed in the GO C–O peak (from 1046 to 1040 cm^−1^) and the berberine methoxy C–O peak (from 1145 to 1142 cm^−1^) suggest the presence of intermolecular interactions between the two components. Importantly, no new covalent bond peaks are observed in the BBR@GO spectrum. This confirms that berberine was loaded onto the GO surface through non-covalent π–π stacking and electrostatic interactions between the planar aromatic structure of berberine and the sp^2^ domains of GO [39].

#### 3.1.2. Thermogravimetric Analysis

The thermal stability of pristine GO and the BBR@GO nanocomposite was evaluated by TGA under nitrogen atmosphere, and the results are presented in Figure 2d. The thermogravimetric curve of pristine GO exhibits a characteristic multi-stage decomposition profile. An initial weight loss of approximately 5% occurs below 100 °C, corresponding to the evaporation of adsorbed water molecules [40]. A sharp and significant weight loss of approximately 25% is observed between 100 and 200 °C. This major decomposition step is attributed to the thermal degradation of labile oxygen-containing functional groups, including hydroxyl, epoxide, and carboxyl groups [41,42]. A gradual and continuous weight loss occurs above 200 °C, extending to 800 °C. This slow mass reduction is associated with the progressive pyrolysis of the more thermally stable carbon backbone [41]. The total residual mass of GO at 800 °C is approximately 32%.

The thermogravimetric curve of BBR@GO displays a distinctly different decomposition profile compared to pristine GO. The initial water loss below 100 °C is reduced to approximately 2–3%. This lower initial weight loss is attributed to the partial shielding of the hydrophilic GO surface by the adsorbed berberine molecules, which reduces the amount of physisorbed water. The weight loss between 100 and 250 °C is more gradual compared to the sharp drop observed in pristine GO. The broadened mass-loss behavior in this region likely reflects overlapping decomposition of GO functionalities and BBR-related species. A major weight loss of approximately 40% is observed in the 250–500 °C temperature range. This decomposition step is absent in pristine GO and might be attributed to the thermal degradation of berberine molecules loaded on the GO surface [43,44]. The total residual mass of BBR@GO at 800 °C is approximately 42%.

The distinct thermal decomposition stage observed between 250 and 500 °C in the BBR@GO curve provides strong thermal evidence for the successful loading of berberine onto the GO nanosheets. The difference in the decomposition profiles between GO and BBR@GO further confirms that the berberine molecules are intimately associated with the GO surface. The higher residual mass of BBR@GO suggests the formation of additional carbonaceous char during pyrolysis under N_2_ [45,46,47].

The berberine fraction was calculated from TGA using the residue mixing rule:

(1) $$\%BBR=\;\frac{R_{BBR@GO}-\;R_{GO}}{R_{BBR}-\;R_{GO}}$$

where R denotes the residual mass (%) at the selected high temperature (800 °C) for GO, BBR, and BBR@GO.

Using R*_GO_* = 32%, R*_BBR@GO_* = 42%, and R*_BBR_* = 48%, the berberine content in BBR@GO is estimated to be ~62.5% (*w*/*w*). This corresponds to ~167% loading relative to GO. GO is known to accommodate high organic uptake via noncovalent interactions. For example, Yang et al. [48] reported doxorubicin loading of ~70% (*w*/*w*) (and 235% loading relative to GO) in the GO–DOX hybrid. However, the present value should be reported as an estimate, since GO–BBR interactions may affect char/residual yields and introduce some uncertainty in residue-based quantification.

#### 3.1.3. SEM-EDS Analysis

**Figure 3:**
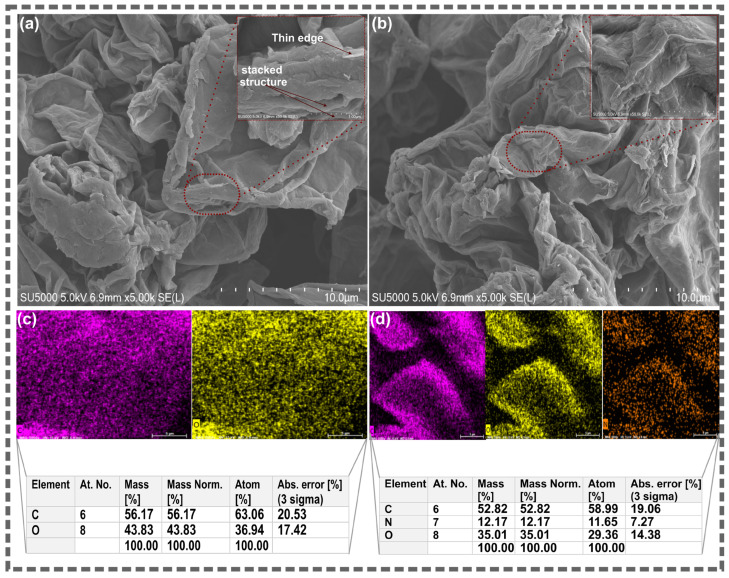
SEM and EDS analysis of (**a**,**c**) GO and (**b**,**d**) BBR@GO.

The morphology and elemental composition of pristine GO and the BBR@GO nanocomposite were examined by SEM-EDS, and the results are presented in Figure 3. The SEM micrograph of pristine GO (Figure 3a) reveals a characteristic layered structure, with a sheet-like morphology. The sheets appear wrinkled and folded, which is typical of graphene oxide produced by chemical oxidation methods [32,42]. The high-magnification inset reveals the thin, layered edges of the GO sheets, where individual layers and stacked multi-layer regions are clearly visible. The SEM micrograph of the BBR@GO nanocomposite (Figure 3b) shows that the overall sheet-like morphology of GO is preserved after berberine loading. This confirms that the non-covalent loading process did not destroy or significantly alter the structural integrity of the GO nanosheets.

The EDS elemental maps of pristine GO (Figure 3c) show uniform distributions of carbon (C) and oxygen (O) across the sheet area. These two elements are consistent with the expected composition of graphene oxide. The EDS elemental maps of BBR@GO (Figure 3d) reveal the presence of a third element, nitrogen (N), in addition to carbon and oxygen. The appearance of nitrogen is the most definitive chemical evidence for berberine loading, as nitrogen is absent in pristine GO and is exclusively contributed by the berberine molecule [44]. The nitrogen signal is uniformly distributed across the GO sheets. This homogeneous distribution confirms that berberine is uniformly loaded across the GO surface rather than concentrated in isolated clusters.

The quantitative EDS analysis provides further compositional evidence. The elemental composition of pristine GO consists of 56.17 wt.% carbon (63.06 at.%) and 43.83 wt.% oxygen (36.94 at.%), yielding a C/O atomic ratio of 1.71. This ratio is consistent with a high degree of oxidation [49,50]. The elemental composition of BBR@GO consists of 52.82 wt.% carbon (58.99 at.%), 12.17 wt.% nitrogen (11.65 at.%), and 35.01 wt.% oxygen (29.36 at.%). The substantial nitrogen content of 12.17 wt.% provides quantitative evidence for berberine incorporation. The C/O atomic ratio increases from 1.71 in GO to 2.01 in BBR@GO. This increase reflects the contribution of the carbon-rich berberine molecules to the overall composition. The decrease in relative oxygen content from 43.83 to 35.01 wt.% is consistent with the dilution effect caused by the addition of berberine, which has a lower O/C ratio than GO.

### 3.2. Adhesion and Coating Thickness

**Figure 4:**
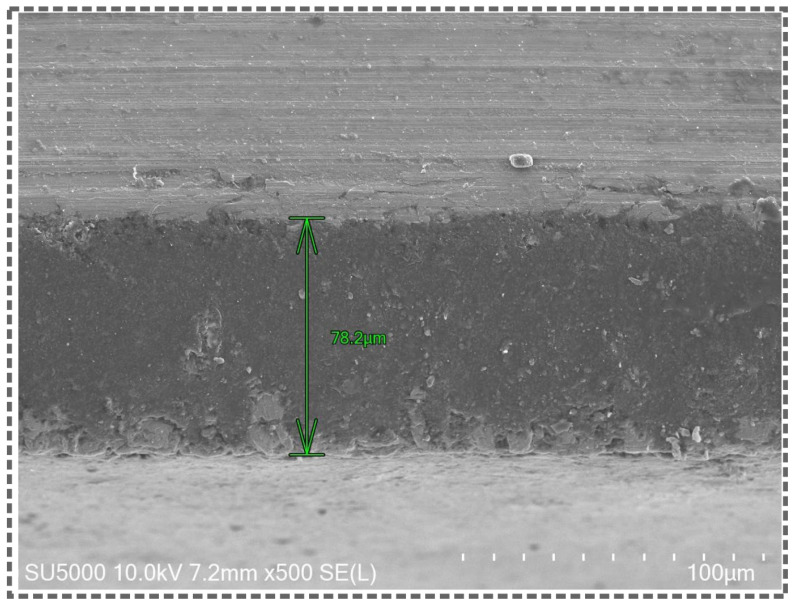
Cross-sectional SEM image of the BBR@GO/EP coating on copper, showing a dense and uniform coating layer with a thickness of approximately 78.2 μm.

The adhesion performance of the BBR@GO/EP coating on the copper substrate was evaluated using the cross-cut tape test in accordance with ASTM D3359, Method B. The coating exhibited excellent adhesion, achieving the highest classification of 5B, with no observable peeling, cracking, or delamination after tape removal. The average thickness of the BBR@GO/EP coating was measured to be 75 ± 5 μm, demonstrating good uniformity across the coated surface. A representative cross-sectional SEM image is presented in Figure 4, which reveals a dense and continuous coating layer with a thickness of approximately 78.2 μm, in close agreement with the average value.

### 3.3. Corrosion Protection Studies

**Figure 5:**
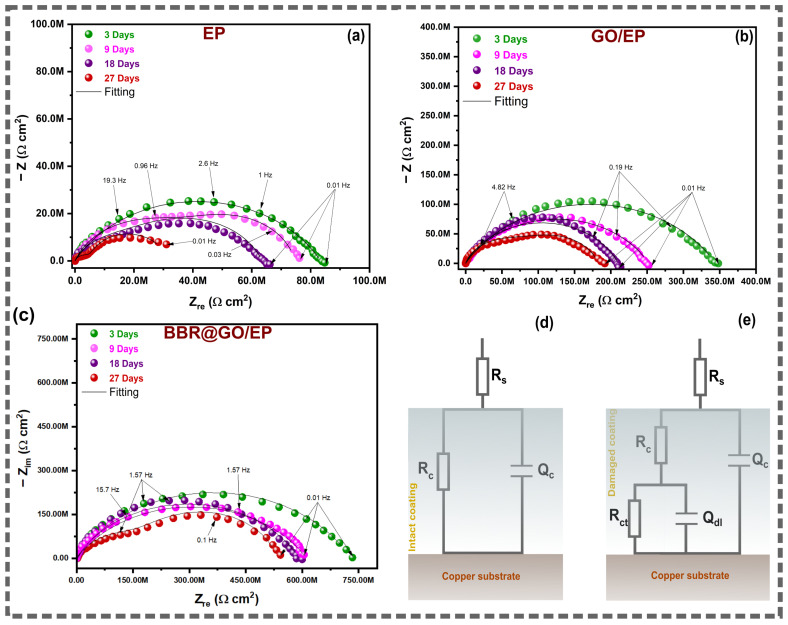
Time-dependent EIS spectra of epoxy-based coatings on copper during immersion in 3.5 wt.% NaCl solution: (**a**–**c**) Nyquist plots of EP, GO/EP, and BBR@GO/EP. (**d**) and (**e**) are the proposed equivalent electrical circuits used for numerical fitting of the EIS data for intact and damaged coatings, respectively.

**Figure 6:**
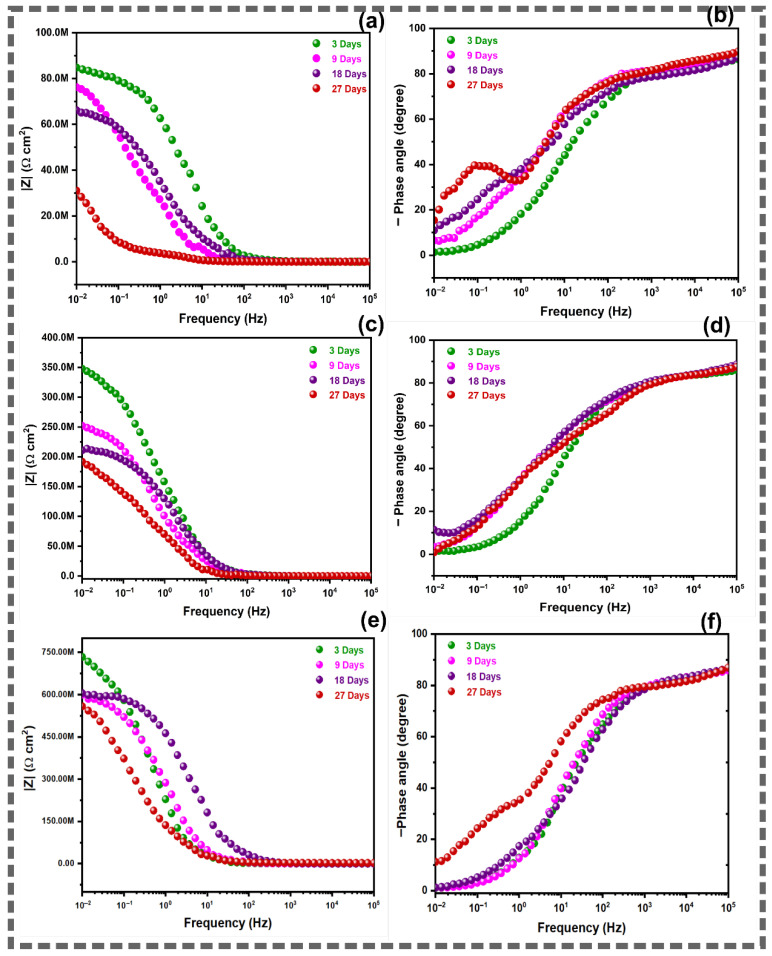
Time-dependent Bode modulus and phase angle plots of epoxy-based coatings on copper during immersion in 3.5 wt.% NaCl solution: (**a**,**c**,**e**) Bode modulus and (**b**,**d**,**f**) phase angle plots of EP (**a**,**b**), GO/EP (**c**,**d**), and BBR@GO/EP (**e**,**f**).

Electrochemical impedance spectroscopy was employed to monitor the corrosion protection performance of the coated copper substrates during prolonged immersion in 3.5 wt.% NaCl solution. The measurements were performed after 3, 9, 18, and 27 days of immersion. The Nyquist plots, Bode impedance modulus plots, and Bode phase angle plots for EP, GO/EP, and BBR@GO/EP coatings are presented in Figure 5 and Figure 6.

#### 3.3.1. Qualitative Analysis of EIS Spectra

The Nyquist plots of the EP coating (Figure 5a) show a single capacitive semicircle at day 3. The diameter of this semicircle is approximately 85 MΩ·cm^2^. A second semicircle appears at low frequencies from day 9 onward. This indicates the onset of electrolyte penetration to the metal–coating interface. The total impedance decreases progressively with immersion time. It reaches approximately 35 MΩ·cm^2^ at day 27. The Bode phase angle plot (Figure 6b) confirms this transition. A single broad peak near −85° is observed on day 3. Two distinct peaks emerge from day 9. The appearance of a second time constant is an indication of coating degradation and the initiation of corrosion reactions at the copper substrate [17,51].

The Nyquist and Bode impedance modulus plots of the GO/EP coating (Figure 5b and Figure 6c) display a significantly larger capacitive semicircle compared to the pure EP coating. The impedance modulus at 0.01 Hz (|Z|_0.01Hz_) is widely used as a quantitative indicator of coating performance [51]. The initial impedance modulus at 0.01 Hz on day 3 is approximately 350 MΩ·cm^2^. This value is 4.4 times higher than that of the pure EP coating. The GO/EP coating maintains a single semicircle up to day 18. A slight depression at low frequencies appears on day 27. This indicates that the electrolyte reaches the metal–coating interface much later than in the pure EP system. The Bode phase angle plot (Figure 6d) shows a single peak close to −83° at day 3. The peak broadens slightly on day 27. This delayed appearance of the second time constant confirms the enhanced barrier effect of GO nanosheets dispersed in the epoxy matrix [16,52].

The Nyquist plots of the BBR@GO/EP coating (Figure 5c) exhibit the largest capacitive semicircles among all three systems. According to Bode modulus plots in Figure 6e, the initial impedance modulus at day 3 is approximately 750 MΩ·cm^2^ at |Z|_0.01Hz_. This value is approximately 9 times higher than that of the pure EP coating and 2 times higher than that of the GO/EP coating. The BBR@GO/EP coating maintains a single, well-defined semicircle up to day 18. A very slight depression appears only on day 27. The Bode phase angle plot (Figure 6f) shows a single peak near −87° at day 3. This value is close to the ideal capacitive response of −90°. These results demonstrate the superior and prolonged barrier protection provided by the BBR@GO nanocomposite.

**Figure 7:**
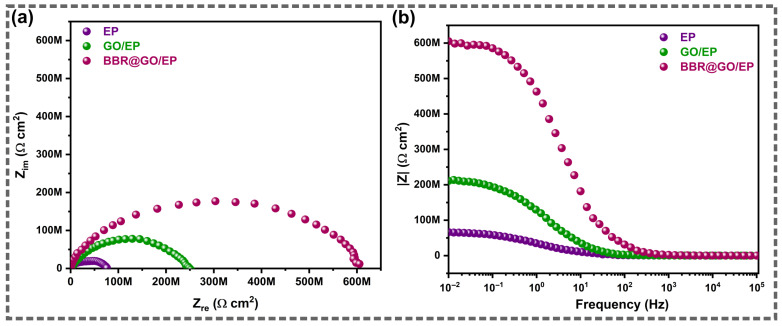
Direct comparison of (**a**) Nyquist and (**b**) Bode modulus (|Z|) plots for EP, GO/EP, and BBR@GO/EP coatings on copper after 18 days of immersion in 3.5 wt.% NaCl.

Overall, the Nyquist and Bode impedance modulus plots (Figure 7) enable a direct comparison of the coating protection level at 18 days of immersion. At this time, BBR@GO/EP shows the largest Nyquist capacitive loop and the highest |Z|_0.01Hz_ across the frequency range, confirming the strongest barrier effect and corrosion resistance. The protection ranking is BBR@GO/EP > GO/EP > EP, in agreement with the fitted resistances in Table 1. The BBR@GO/EP coating exhibits an initial |Z|_0.01Hz_ of approximately 750 MΩ·cm^2^ at day 3. This value decreases to approximately 560 MΩ·cm^2^ at day 27. The GO/EP coating shows values of 350 and ~200 MΩ·cm^2^ at days 3 and 27, respectively. The pure EP coating shows the lowest values of 85 and 35 MΩ·cm^2^ at days 3 and 27, respectively.

#### 3.3.2. Equivalent Electrical Circuit Models and EIS Parameters

The EIS data were analyzed using equivalent electrical circuit (EEC) models to extract quantitative electrochemical parameters. Two models were employed depending on the stage of coating degradation [52,53].

For intact coatings exhibiting a single time constant, a one-time-constant model was used (Figure 5d). This model consists of the solution resistance (R_s_) in series with a parallel combination of the coating constant phase element (CPE_c_) and the coating resistance (R_c_). In this model, R_c_ represents the resistance of the coating to ionic transport. A high R_c_ value indicates a dense, defect-free coating with low porosity [52,54].

For degraded coatings exhibiting two-time constants, a two-time-constant model was used. This model adds a second parallel combination at the metal–coating interface (Figure 5e). In this model, R_ct_ represents the charge transfer resistance at the metal surface. Q_dl_ represents the constant phase element of the electrical double layer formed at the metal–electrolyte interface beneath the coating [54,55]. The appearance of the second time constant indicates that the electrolyte has permeated through the coating and reached the metal substrate, initiating electrochemical corrosion reactions [52].

A CPE was used instead of an ideal capacitor to account for the non-ideal capacitive behavior arising from surface roughness, heterogeneity, and distributed reactivity [56,57]. The impedance of a CPE is defined as:

(2) $$Z_{CPE}(\omega )=\;\frac{1}{{\left(j\omega \right)}^{n}Q}$$

where Q is the CPE parameter, ω is the angular frequency (in rad s^−1^), j is the imaginary unit, and n is the CPE exponent (0 ≤ n ≤ 1). When n = 1, the CPE reduces to an ideal capacitor. When n = 0, it reduces to a pure resistor [56].

Brug’s formula was employed to determine the effective capacitance of the porous film layer described by the constant phase element [58,59]:

(3) Ceff=Qdl(1ndl)RsRctRs+Rct1−nn

This formula is appropriate for the double-layer CPE because the CPE behavior at the metal–electrolyte interface arises from a surface (lateral) distribution of time constants [58,59]. This distribution is caused by the heterogeneous nature of the corroding metal surface beneath the coating [59]. The fitted EIS parameters for all coatings at different immersion times are summarized in Table 1.

The coating resistance (R_c_) is a direct measure of the coating’s ability to resist ion transport. A high R_c_ value indicates a superior barrier property [55]. At day 3, the R_c_ value for the BBR@GO/EP coating was 728.9 MΩ·cm^2^. This is 8.7 times higher than the R_c_ for the pure epoxy coating (83.3 MΩ·cm^2^) and 2.1 times higher than for the GO/EP coating (348.3 MΩ·cm^2^). This demonstrates the significantly enhanced initial barrier performance due to the incorporation of the BBR@GO nanocomposite. The GO nanosheets create a tortuous pathway for electrolyte diffusion, while the berberine molecules can improve the filler-matrix adhesion [18].

The CPE exponent for the coating (n_c_) provides information about its homogeneity. A value of n_c_ close to 1 indicates a more uniform, ideal dielectric behavior [56]. A decrease in n_c_ over time reflects an increase in surface heterogeneity and degradation. The pure EP coating showed the largest decrease in n_c_, from 0.7914 to 0.6928. This indicates structural degradation. The BBR@GO/EP coating maintained the most stable and highest n_c_ values, decreasing by only 0.0233 over 27 days. This confirms that the BBR@GO/EP coating retains its structural integrity and homogeneity most effectively.

The appearance of a second time constant in the EIS spectra indicates that the electrolyte has reached the metal substrate and corrosion has initiated [52]. For the pure EP coating, this occurred on day 9. For the GO/EP and BBR@GO/EP coatings, this was delayed until day 27. This delay demonstrates their superior barrier properties.

The charge transfer resistance (R_ct_) is inversely proportional to the corrosion rate at the metal/coating interface [55]. At day 27, the R_ct_ for the BBR@GO/EP coating was 297.7 MΩ·cm^2^. This value is 12.0 times higher than for the pure EP coating (24.8 MΩ·cm^2^) and 3.7 times higher than for the GO/EP coating (79.9 MΩ·cm^2^). The high R_ct_ for the BBR@GO/EP system indicates a very low corrosion rate. This may be attributed to the effect of berberine molecules on the GO dispersion and/or interfacial contact with the metal, which block active corrosion sites on the copper surface [16,60].

The effective double layer capacitance (C_dl,eff_) is related to the area of the metal surface exposed to the electrolyte. A lower C_dl,eff_ value suggests a smaller delaminated area and less corrosion [16,46]. At day 27, the C_dl,eff_ for the BBR@GO/EP coating (2.95 × 10^−9^ F·cm^−2^) was 45 times lower than for the pure EP coating (1.32 × 10^−7^ F·cm^−2^). This confirms that the BBR@GO/EP coating significantly minimizes interfacial delamination and corrosion. Overall, the EIS results provide a comprehensive and quantitative confirmation of the improved corrosion protection performance of the BBR@GO/EP coating. It exhibits the highest barrier resistance, the most stable structure, and the most effective inhibition of interfacial corrosion over the 27-day immersion period.

### 3.4. Potentiodynamic Polarization

**Figure 8:**
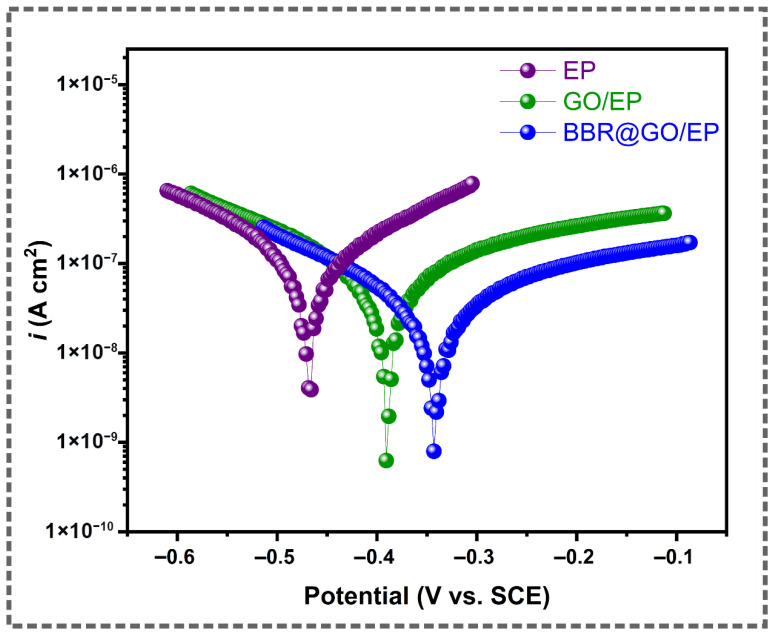
Potentiodynamic polarization curves for the coated samples after 27 days of immersion in 3.5 wt.% NaCl solution.

Potentiodynamic polarization measurements were conducted to further investigate the corrosion protection behavior of the coatings. The tests were performed after 27 days of immersion in 3.5 wt.% NaCl solution. Figure 8 shows the Tafel polarization curves for the EP, GO/EP, and BBR@GO/EP coated copper samples.

The key electrochemical parameters were extracted from the Tafel plots and listed in Table 2. These parameters include the corrosion potential (E_corr_), corrosion current density (i_corr_), anodic Tafel slope (β_a_), and cathodic Tafel slope (β_c_).

The pure epoxy coating exhibited a corrosion potential of −0.468 V vs. SCE. It also showed the highest corrosion current density of 1.04 × 10^−7^ A cm^−2^. The incorporation of GO nanosheets shifted the E_corr_ to a more positive value of −0.387 V vs. SCE. The *i*_corr_ was reduced to 6.32 × 10^−8^ A cm^−2^. This demonstrates a significant improvement of protection. The positive shift in E_corr_ suggests that the GO nanosheets primarily act by blocking anodic sites on the copper surface. This restricts the dissolution of copper metal.

The BBR@GO/EP coating showed the most positive E_corr_ of −0.338 V vs. SCE. This represents a significant anodic shift relative to the pure epoxy coating. The *i*_corr_ was also reduced to 2.59 × 10^−8^ A cm^−2^. Both the anodic and cathodic current densities were significantly suppressed compared to the pure epoxy. This indicates the suppression of both partial corrosion reactions. The GO nanosheets reinforced by BBR molecules provide a strong protective barrier. This explains the superior performance observed in the PDP results compared to EP and GO/EP.

### 3.5. Surface Morphology After Immersion

**Figure 9:**
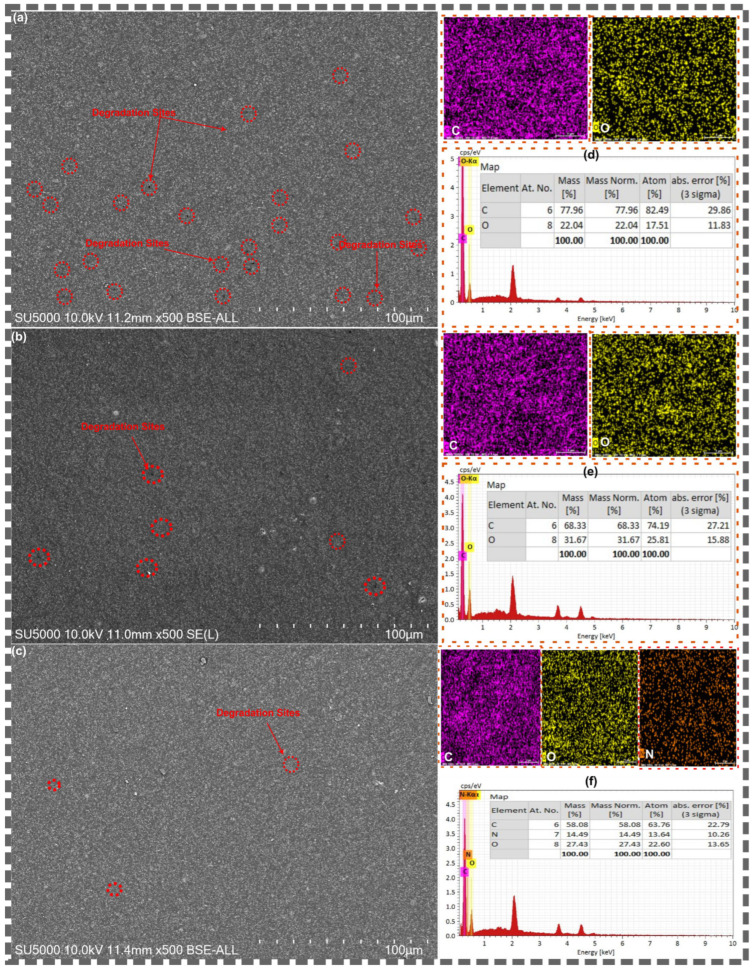
SEM-EDS analysis of (**a**,**d**) EP, (**b**,**e**) GO/EP, and (**c**,**f**) BBR@GO/EP after 27 days of immersion in 3.5 wt.% NaCl.

The surface morphology of the coatings was examined by SEM after 27 days of immersion in 3.5 wt.% NaCl solution. The results are presented in Figure 9. The SEM micrograph of the pure epoxy coating is shown in Figure 9a. The surface exhibits extensive degradation. Numerous pore-like degradation sites are visible across the entire area. This indicates significant structural damage to the coating. The SEM micrograph of the GO/EP coating is shown in Figure 9b. The surface shows a reduced number of degradation sites compared to the pure EP coating. The overall surface integrity is improved. However, some defects are still present. This suggests that the GO nanosheets provide a partial barrier against degradation but do not completely prevent it. The SEM micrograph of the BBR@GO/EP coating is shown in Figure 9c. The surface appears remarkably smooth and intact. Very few defects or degradation sites are observed. This visual evidence demonstrates the good durability and barrier integrity of the BBR@GO/EP coating after prolonged exposure to the corrosive environment.

EDS analysis was performed to investigate the elemental composition of the coatings after immersion. The results are shown in Figure 9d–f. The EDS spectrum of the pure EP coating (Figure 9d) and the GO/EP coating (Figure 9e) detected carbon and oxygen. The EDS spectrum of the BBR@GO/EP coating (Figure 9f) detected carbon, oxygen, and nitrogen. The presence of a significant nitrogen signal (14.49 wt.%) confirms the retention of berberine within the coating matrix even after 27 days of immersion. The elemental maps show a uniform distribution of nitrogen across the surface. This indicates that the BBR@GO nanocomposite remains well-dispersed and stable within the epoxy matrix. The excellent surface integrity and the confirmed retention of the inhibitor are consistent with the observed corrosion protection performance demonstrated in electrochemical tests.

### 3.6. FTIR Analysis of Coatings After Immersion

**Figure 10:**
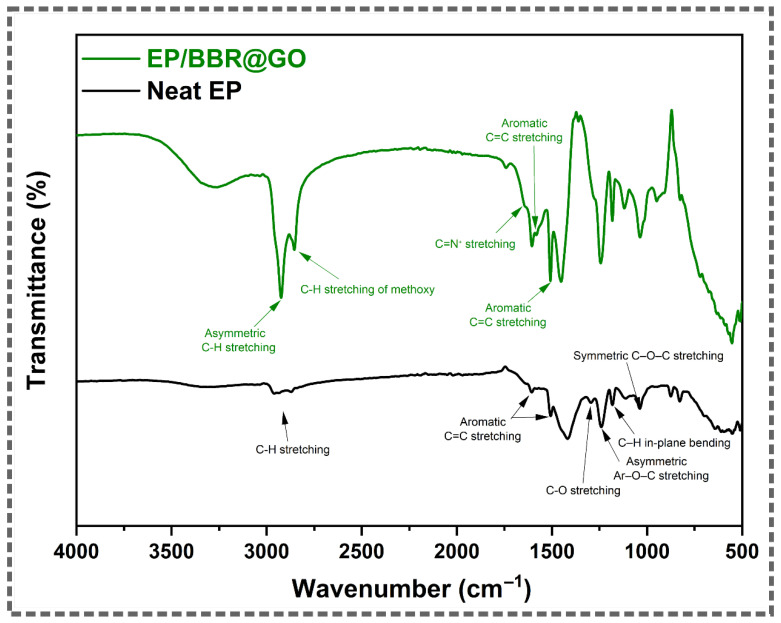
FTIR spectra of the neat epoxy and the BBR@GO/EP coatings after the immersion test.

FTIR spectroscopy was used to analyze the chemical stability of the coatings after 27 days of immersion in 3.5 wt.% NaCl solution. Figure 10 shows the FTIR spectra of the neat epoxy and the BBR@GO/EP coatings after the immersion test.

The spectrum of the neat EP coating shows the characteristic absorption bands of a cured epoxy resin. The main peaks include C–H stretching at approximately 2920 cm^−1^, aromatic C=C stretching at 1608 and 1508 cm^−1^, and C–O–C ether stretching at 1245 and 1035 cm^−1^ [16,17]. The absence of the oxirane ring peak at 915 cm^−1^ confirms the complete curing of the epoxy matrix. The preservation of these peaks after 27 days indicates that the main polymer backbone remains chemically stable in the corrosive environment.

The spectrum of the BBR@GO/EP coating displays a clear superposition of the peaks from both the epoxy matrix and the BBR@GO nanocomposite. In addition to the epoxy backbone peaks, the diagnostic peaks of berberine are visible. The C=N^+^ stretching vibration of the iminium group is present at 1635 cm^−1^ [34]. The C–H stretching of the methoxy groups is observed at 2844 cm^−1^ [34,35]. The persistence of these characteristic berberine peaks after 27 days of immersion is an important finding. It confirms that the berberine is successfully retained within the coating matrix and does not leach out into the solution. This result is consistent with the EDS analysis, which also detected nitrogen on the coating surface after the immersion test. The chemical stability of the BBR@GO filler and its strong non-covalent anchoring to the GO nanosheets are responsible for this excellent retention. This sustained presence of the berberine molecule within the coating is essential for providing effective corrosion protection at the coating–metal interface.

### 3.7. Benchmarking Against Previous GO/Epoxy Coatings

To contextualize the performance of the BBR@GO/EP coating, a systematic comparison was conducted with recently reported GO-modified epoxy systems, using the impedance modulus at 0.01 Hz (|Z|_0.01_ Hz) as a key indicator of barrier performance. Table 3 summarizes the relevant EIS parameters, including substrate, coating thickness, electrolyte, immersion time, and initial and final |Z|_0.01_ Hz values.

The results indicate that the BBR@GO/EP coating occupies a competitive position among GO-based epoxy systems. Some studies reported clearly superior performance. Zhou et al. [61] achieved an initial |Z|_0.01_ Hz of ~10^11^ Ω·cm^2^ using a GO-hybridized waterborne epoxy on steel. Xu et al. [62] reported a final |Z|_0.01_ Hz of 2.23 × 10^10^ Ω·cm^2^ after 70 days with dual-functionalized GO, while Zhang et al. [63] maintained 3.05 × 10^9^ Ω·cm^2^ after 100 days using a ZnAl-LDH/GO composite. These systems commonly employed steel substrates, multifunctional fillers, and optimized coating architectures. Other coating systems exhibited comparable performance. Feng et al. [64], Wu et al. [65], and Qiang et al. [66] reported |Z|_0.01_ Hz values in the range of 10^8^–10^9^ Ω·cm^2^ over relatively short immersion periods. Notably, the present coating achieves similar protection on copper, which is more difficult to protect than steel due to its higher electrochemical nobility and distinct corrosion behavior [67].

Hu et al. [68] and Lyu et al. [69] reported final |Z|_0.01_ Hz values between 10^5^ and 10^8^ Ω·cm^2^, lower than those obtained in this work. These coatings relied also on functionalized GO, highlighting the excellent effect of combining GO with berberine compared to several previously reported materials.

It should be noted that direct comparison among studies remains approximate due to variations in substrate, coating thickness, filler content, curing conditions, and electrolyte composition [70]. Nevertheless, the present results demonstrate that the BBR@GO/EP coating provides protection that is competitive with, and in many cases superior to, previously reported GO-modified epoxy systems, particularly considering its straightforward synthesis process. However, there are several directions for further improvement. First, optimization of coating thickness and GO loading could yield a different performance. Second, the introduction of other “green” molecules for GO functionalization along with hybrid 2D materials may improve interfacial compatibility and reduce defect density. Finally, extending immersion testing beyond 27 days would enable a more comprehensive assessment of long-term durability.

**Table 3:** Comparison of EIS performance metrics for various GO-modified epoxy coatings.

Coating System	Substrate	Thickness (μm)	Electrolyte	Duration (Days)	Initial |Z| (Ω·cm^2^)	Final |Z| (Ω·cm^2^)	Reference
GO-hybridized waterborne epoxy (GOWE)	Steel	60	3.5 wt.% NaCl	64	~10^11^	9.7 × 10^9^	[61]
Vanillin + organosilicon functionalized GO/VER	Steel	50	3.5 wt.% NaCl	70	1.21 × 10^11^	2.23 × 10^10^	[62]
ZnAl-LDH/GO/epoxy	Q235 steel	158.6–163.4	3.5 wt.% NaCl	15–100	3.71 × 10^10^	3.05 × 10^9^	[63]
BBR@GO/EP	Copper	75	3.5 wt.% NaCl	27	7.5 × 10^8^	5.31 × 10^8^	This work
BTA-SiO_2_-GO/epoxy	Carbon steel	100	3.5 wt.% NaCl	7	9.13 × 10^8^	3.61 × 10^9^	[64]
Bio-based epoxy-GO (GODN1%/EP)	Carbon steel	50	3.5 wt.% NaCl	45	9.2 × 10^9^	4.38 × 10^8^	[65]
GO-Ti_3_C_2_T_x_/epoxy (GO-MXene)	Q235 steel	50	3.5 wt.% NaCl	8	2.72 × 10^9^	1.84 × 10^8^	[66]
SiO_2_/KH570-GO/epoxy (G-S-K)	Q235 steel	30	3.5 wt.% NaCl	10	9.7 × 10^8^	1.9 × 10^6^	[68]
CeO_2_-GO/epoxy	Q235 steel	—	Saline–alkali	31	1.51 × 10^8^	1.12 × 10^7^	[69]

### 3.8. Corrosion Protection Mechanism

**Figure 11:**
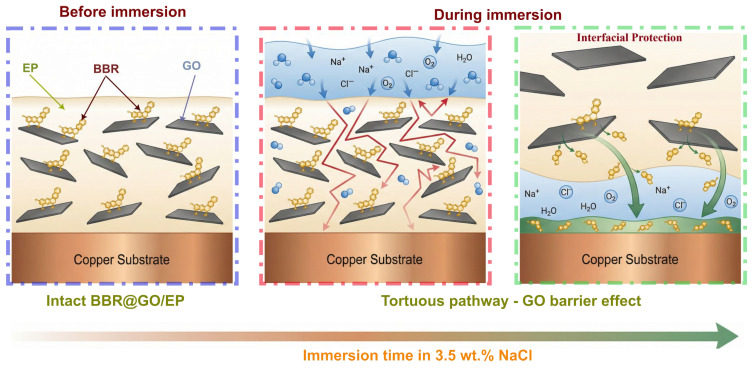
A graphical illustration of the proposed corrosion protection mechanism by BBR@GO/EP.

The BBR@GO/EP coating demonstrated an improved corrosion protection for copper in 3.5 wt.% NaCl for 27 days. Figure 11 provides a schematic illustration of this combined protection model.

The primary defense is the physical barrier created by the well-dispersed GO nanosheets. These impermeable, high-aspect-ratio sheets establish a tortuous diffusion pathway within the epoxy matrix [17]. This “labyrinth effect” significantly lengthens the path that corrosive species like water, oxygen, and chloride ions must travel to reach the copper substrate [21,71]. Our results confirm this enhanced barrier function. The BBR@GO/EP coating showed a significantly higher initial coating resistance (R_c_) and delayed the onset of interfacial corrosion compared to the neat epoxy. Complementing this physical barrier is the effect of the berberine molecules. The presence of BBR throughout the coating, and particularly at the interface, would improve the GO dispersion and might contribute to coordination with the substrate. The slow permeation of electrolyte due to the GO barrier creates a more stable environment at the interface. This might allow the BBR molecules to effectively adsorb onto the copper surface and form a protective film [72,73]. Importantly, post-immersion EDS and FTIR analyses confirmed the retention of BBR within the coating, which is essential for this long-term effect.

Overall, the synergy between the GO barrier and the BBR is key. The GO nanosheets physically delay the corrosion process, which allows the retained BBR inhibitor to form a stable and highly effective protective barrier at the copper interface, resulting in excellent long-term corrosion resistance.

## 4. Conclusions

A novel anticorrosion coating for copper was successfully developed by incorporating a berberine-loaded graphene oxide (BBR@GO) nanocomposite into an epoxy matrix. The key findings of this study are as follows:

A BBR@GO nanocomposite was successfully synthesized via a simple, non-covalent functionalization method. FTIR, TGA, and SEM-EDS analyses confirmed the uniform loading of berberine onto the GO nanosheets through π-π stacking interactions, without altering the structural integrity of the GO.

The incorporation of 0.1 wt.% BBR@GO into the epoxy matrix significantly enhanced the corrosion protection performance for copper in 3.5 wt.% NaCl solution. The BBR@GO/EP coating demonstrated a total impedance of 5.31 × 10^8^ Ω·cm^2^ after 27 days, which was significantly higher than both the pure epoxy (3.14 × 10^7^ Ω·cm^2^) and the GO-only reinforced epoxy (1.93 × 10^8^ Ω·cm^2^) coatings.

Potentiodynamic polarization results corroborated the EIS findings, showing that the BBR@GO/EP coating reduced the corrosion current density by 75% and shifted the corrosion potential by +130 mV compared to the pure epoxy coating, indicating effective suppression of both anodic and cathodic reactions.

Post-immersion SEM, EDS, and FTIR analyses confirmed the durability of the BBR@GO/EP coating and, crucially, the retention of berberine within the coating matrix after 27 days of exposure. This sustained presence of the inhibitor at the interface is essential for effective protection.

The enhanced performance is attributed to a synergistic mechanism. The well-dispersed GO nanosheets create a tortuous pathway that provides a physical barrier against corrosive species, while the retained berberine molecules provide an inhibitive effect at the coating–metal interface.

This work demonstrates that the non-covalent functionalization of GO with a natural, green inhibitor is a simple and effective strategy for developing high-performance, environmentally friendly anticorrosion coatings for copper substrates.

## Figures and Tables

**Table 1:** Electrochemical impedance parameters obtained by fitting the EIS data of EP, GO/EP, and BBR@GO/EP coatings at different immersion times in 3.5 wt.% NaCl solution.

System	Immersion Time (Days)	Rs (Ω·cm^2^)	Coating Response			Charge Transfer/Double Layer				R_total_ (MΩ·cm^2^)
			Q_c_ (S·s^n^ cm^−2^)	n_c_	R_c_ (MΩ·cm^2^)	Q_dl_ (S·s^n^ cm^−2^)	n_dl_	R_ct_ (MΩ·cm^2^)	C_dl,eff_ (F·cm^−2^)	
EP	3	18.358	1.363 × 10^−9^	0.7914	83.34	—	—	—	—	83.348
	9	24.467	4.018 × 10^−9^	0.7839	46.33	4.317 × 10^−8^	0.8315	30.47	4.120 × 10^−8^	76.803
	18	26.784	8.666 × 10^−9^	0.7811	36.75	7.356 × 10^−8^	0.8118	28.66	7.651 × 10^−8^	65.416
	27	28.245	2.033 × 10^−8^	0.6928	6.57	1.426 × 10^−7^	0.8002	24.78	1.323 × 10^−7^	31.363
GO/EP	3	55.726	6.805 × 10^−10^	0.8710	348.25	—	—	—	—	348.253
	9	60.054	8.289 × 10^−10^	0.8734	252.42	—	—	—	—	252.420
	18	65.874	1.462 × 10^−9^	0.8360	210.36	—	—	—	—	210.362
	27	65.237	1.559 × 10^−9^	0.8214	112.61	1.079 × 10^−8^	0.8045	79.89	9.131 × 10^−9^	192.503
BBR@GO/EP	3	66.224	1.884 × 10^−10^	0.8705	728.89	—	—	—	—	728.892
	9	69.483	6.627 × 10^−10^	0.8645	611.66	—	—	—	—	611.664
	18	73.988	6.559 × 10^−10^	0.8463	591.00	—	—	—	—	591.004
	27	78.109	7.941 × 10^−10^	0.8472	233.33	3.302 × 10^−9^	0.8802	297.73	2.946 × 10^−9^	531.060

**Table 2:** Potentiodynamic polarization parameters for the coated samples after 27 days of immersion.

Coating	E_corr_ ^a^ (V vs. SCE)	*i*_corr_ ^b^ (A cm^−2^)	β_a_ ^c^ (mV dec^−1^)	β_c_ ^d^ (mV dec^−1^)
EP	−0.468	1.04 × 10^−7^	85	100
GO/EP	−0.387	6.32 × 10^−8^	72	115
BBR@GO/EP	−0.338	2.59 × 10^−8^	62	118

^a^ The standard deviation range for E_corr_: [3.7–5.9%]. ^b^ The standard deviation range for i_corr_: [4.8–6.2%]. ^c^ The standard deviation range for β_a_: [5.3–6.1%]. ^d^ The standard deviation range for β_c_: [3.3–7.2%].

## Data Availability

The original contributions presented in this study are included in the article. Further inquiries can be directed to the corresponding author.
